# Supplementary figures and images for: Complete Chloroplast Genomes of 14 Subspecies of D. glomerata: Phylogenetic and Comparative Genomic Analyses
Source: Genes (Basel). 2022 Sep 9;13(9):1621. doi: 10.3390/genes13091621 (PMC9498378; doi:10.3390/genes13091621)

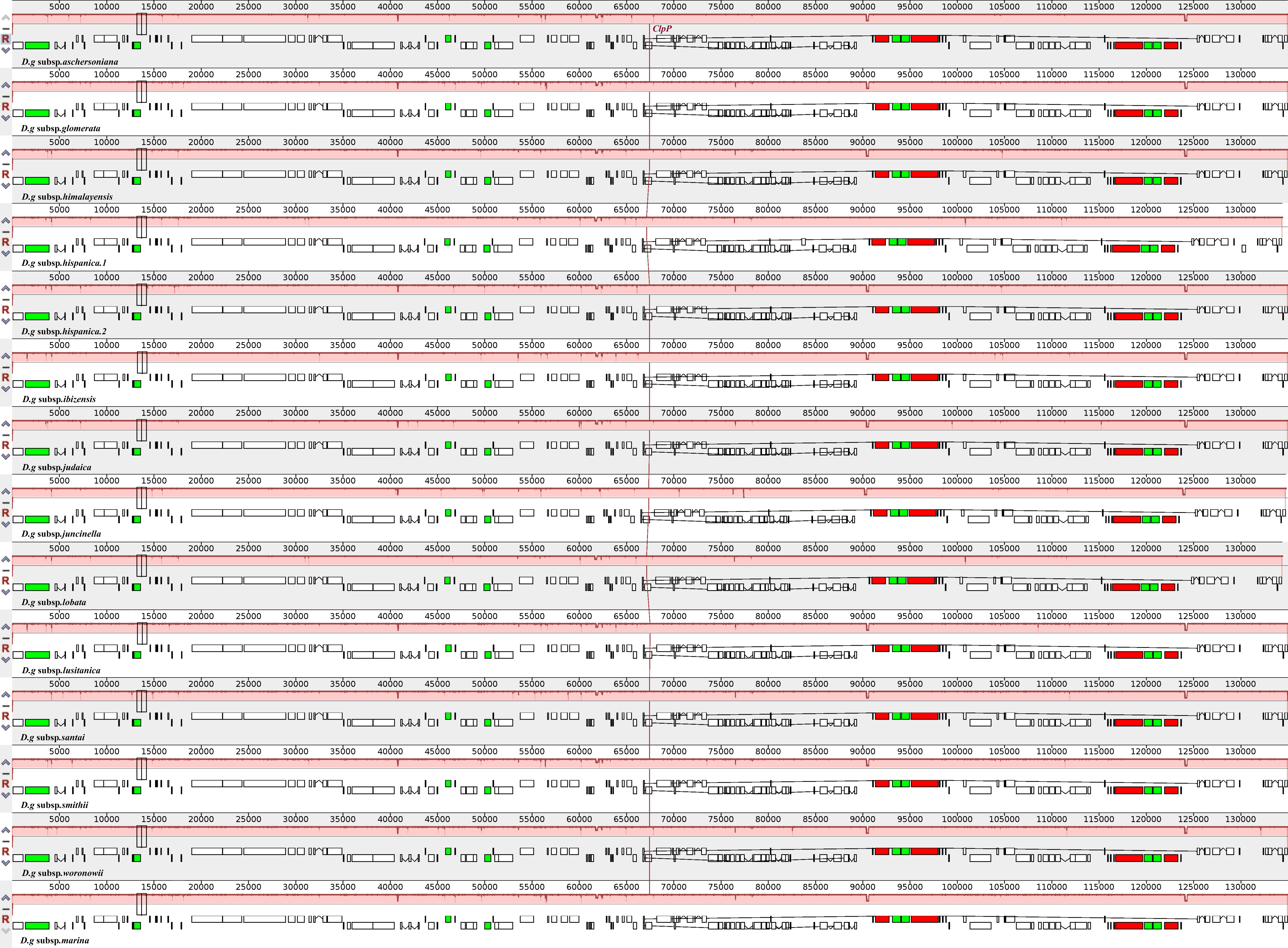

Supplement: Supplementary file 1 [file genes-13-01621-s001.zip › Figure S1.png]
